# Characteristics of emergency nurse practitioner professional identity: A multicenter qualitative study

**DOI:** 10.1016/j.ijnsa.2025.100384

**Published:** 2025-07-16

**Authors:** Aline Chenou, Loïc Brillouet, Stéphane Guillon, Pascal Bilbault, Thierry Pelaccia

**Affiliations:** a Emergency Department, Strasbourg University Hospital F-67200 Strasbourg, France; b Hospital Center of Rouffach F-68250 Rouffach, France; c Laboratoire Interuniversitaire des Sciences de l’Éducation et de la Communication (LISEC), UR 2310, University of Strasbourg F-67000 Strasbourg, France; d INSERM (French National Institute of Health and Medical Research), UMR 1260, Regenerative NanoMedicine (RNM), Fédération de Médecine Translationnelle (FMTS), University of Strasbourg, Strasbourg, France; e Prehospital Emergency Care Service (SAMU 67), Strasbourg University Hospital F-67200 Strasbourg, France; f Centre for Training and Research in Health Sciences Education (CFRPS), Faculty of Medicine, University of Strasbourg F-67000 Strasbourg, France

**Keywords:** Advanced practice nurses, Professional role, Nursing identity, Scope of practice, Professional identity, Emergency Department, Leadership in Nursing, Qualtitative Research

## Abstract

**Context:**

The first emergency nurse practitioners have been working in France for two years now. Their arrival may raise questions, but they are seen as a potential solution to the crisis of overcrowding in emergency department. As a new profession in the emergency department, it is crucial to study the characteristics of the professional identity in order to provide the keys to understanding their place and the role they can play within systems of care.

**Aims:**

We wanted to explore the perceived characteristics of the emergency nurse practitioners by the physicians, nurses, health care managers, and emergency nurse practitioners themselves

**Methods:**

This is a multicenter qualitative study. 21 semi-structured interviews were conducted in 5 distinct hospitals from April to June 2024. Participants included nurses, emergency nurse practitioners, physicians, and healthcare managers. Data were analyzed using thematic coding.

**Results:**

Four themes emerged concerning the characteristics of emergency nurses practitioners professional identity: A combination of skills in a variety of fields (1), a pivotal professional, expert in the care pathway, at the interface with other emergency professions (2), a dual identity anchor: nurse and emergency professional, (3) and a position that is still in its nascent stages, plagued by uncertainty and obstacles (4).

**Conclusion:**

This study revealed the characteristics of the professional nursing identity. It emerges that professionals see the emergency nurse practitioners as an intermediary player in the care response and as an expert in the care pathway.

**Contribution of the paper**

**What is already known?**

• Advanced practice is well developed in some countries, but in others, it is just beginning.

• In France, emergency nurse practitioners began working in 2021 but their self-definition is unclear.

• A global crisis in emergency services is struggling to find viable solutions.

**What this paper add:**

• The emergency nurse practitioner is recognize as an expert in the patient pathway

• The emergency nurse practitioner appears to have a hybrid professional identity that is in need of further investigation and could be defined by the new concept of 'mesopraxia'.

• The emergency nurse practitioner could be part of the answer to the overcrowding emergency crisis.

Alt-text: Unlabelled box

## 1 Introduction

While advanced practice nursing has long been established in some countries, it is now gaining ground around the world, not least in France, where it was born in 2018. The advent of advanced practice nurses in France is intended to enhance the availability of healthcare services and foster the development of nursing research and knowledge production.

In a context of overcrowding in emergency room (ER), emergency nurse practitioners appeared in France in 2021. Three years later, approximately thirty emergency nurse practitioners are practicing in France, and this number is expected to triple in 2025 (Tiberti et al., 2024). While the social representations surrounding the identity of nurse have been the subject of research (Fagermoen, 1997; Hoeve et al., 2014), to our knowledge, the specificities of the emergency designation have not yet been explored.

Professional identity is often seen as the adoption of a profession's values, norms, and ethical standards. Some researchers argue that this identity shapes nurses' daily clinical practice (Fagermoen, 1997). There has been extensive discussion about nurses' professional identity. Although Florence Nightingale viewed nursing as a distinct profession, its nature is often unclear to the public and frequently linked with the medical field (Hoeve et al., 2014).

It's crucial to note that defining professional identity is vital for a profession's academic growth (Arthur and Randle, 2007). Additionally, new theoretical concepts that highlight the unique aspects of nursing professional identity are essential to scientifically support policies(Johnson et al., 2012).

The ER is a unique hospital setting that has faced an international crisis for a long time (Di Somma et al., 2015). This ongoing crisis lacks a lasting solution, as most countries are currently dealing with significant ER overcrowding. The ER crisis is a major public health issue, affecting both patients and healthcare systems (Roussel et al., 2023). While there is strong political hope that emergency nurse practitioners will help resolve this crisis, opinions among those in the field vary widely. These views range from seeing emergency nurse practitioners as super-nurses or mini-physicians, and sometimes reflect concerns about the rise of low-cost medicine.

In a world where the majority of health care systems are experiencing significant challenges, and where advanced practice nursing is viewed as a potential solution to ensuring continued access to high-quality care, it is crucial to examine the characteristics of the professional identity of emergency nurse practitioners. Indeed, a recent review of the literature has highlighted the necessity to examine the role of advanced practice nurses in ERs (Horvath et al., 2023). It should be noted that in France, emergency nurse practitioners are defined as advanced practice nurses who have received training at a master's level to intervene in a variety of clinical situations within emergency settings. The professional identity of these individuals is shaped by a combination of advanced clinical expertise, autonomous decision-making within defined organizational frameworks, and collaborative work alongside physicians and multidisciplinary teams. Emergency nurse practitioners are able to operate within two distinct care pathways. The first is a collaborative pathway, in which they contribute to the management of complex patients under a physician's diagnostic and therapeutic guidance. The second allows greater autonomy, enabling the emergency nurse practitioner to independently manage certain patient presentations, provided a physician is involved at some point during the care process. Emergency nurse practitioners are distinguished from other advanced practice nursing specializations by virtue of their involvement in the management of acute and decompensated conditions. Despite the extension of direct patient access to all advanced pracrice nurses specializations in 2025, emergency nurse practitioners maintain a distinctive position within the French context, as they are authorized to manage life-threatening conditions in collaboration with physicians. Historically, emergency nurse practitioners in France were limited to the care of patients with chronic diseases.

In both configurations, emergency nurse practitioners are authorized to perform detailed clinical assessments, request and interpret diagnostic tests, and prescribe treatments — either as initial prescriptions or as renewals— from predefined lists of medications and health products established by regulatory authorities. The role of these professionals is of particular significance in the optimization of patient flow, the enhancement of continuity of care, and the support of the management of vulnerable or high-need populations, including older adults, patients with multiple comorbidities, or those facing psychosocial difficulties.

Beyond the provision of direct care, emergency nurse practitioners also make significant contributions to a range of transversal missions, including clinical teaching, quality improvement, protocol development, and applied research. The establishment of this novel profession in French ERs and the investigation of its distinctive identity characteristics, as well as the challenges and opportunities encountered, provides a valuable opportunity for other health systems contemplating the creation of emergency nurse practitioners to anticipate the potential implications and challenges ahead. This research effort can also be framed within an activity analysis approach, one of whose objectives is to reflect on work in order to better understand the conditions of entry into the profession and the development of skills (Barbier, 2019; Mayen, 2022)

From a pedagogical standpoint, this type of research could facilitate students’ ability to situate themselves and comprehend their future professional context. It could also provide trainers with insights and strategies to encourage students' professionalization, and the development of their professional identity.

## 2 Background

Identity is a dynamic cognitive process that encompasses phases of elaboration and maturation, interspersed with crises. Concurrently, it is characterized by a certain degree of consistency, which serves to provide relative coherence, thereby conferring a sense of identity (Dubar, 2007; Mucchielli, 2009)

The concept of identity is one of the most pivotal concepts for situating the individual within a collective. As evidenced by Baudry and Juchs’ historiographical analysis, it serves as a foundational instrument for contemplating the position of an individual within a group, integrating social history and the history of representations (Baudry and Juchs, 2007).

### 2.1 Nursing identity

There are several reasons why it is crucial to understand and work on the concept of identity, one of the first of these is that an identity with clearly delineated contours will ensure longevity in the profession (Johnson et al., 2012).

Nurses’ professional identity is characterized by strong socio-historical aspects (Hoeve et al., 2014; Kasperiuniene and Zydziunaite, 2019) with an ingrained idea of subordination to the medical profession (Hoeve et al., 2014). The influence exerted by the cultural and social context in which nurses evolve must also be emphasized (Fitzgerald, 2020). A review of the literature revealed a gap between nurses' perceptions of their own profession and those of the general public, which may be exacerbated by the media (Hoeve et al., 2014). Nursing suffers from many stereotypes, and nurses are recognized primarily for their virtue, not for their knowledge or as autonomous professionals (Hoeve et al., 2014).

To counteract stereotypes, for a long time, many authors emphasize the need for further research (Fitzgerald, 2020; Johnson et al., 2012; Öhlén and Segesten, 1998). A strong professional identity is a guarantee of homogeneity within a profession and can have a strong influence on policies towards a profession, because identity reflects who professionals become and how they feel connected to each other (van der Cingel and Brouwer, 2021). Indeed, nurses recognize themselves as a distinct professional group with a group entity based on similar or identical pillars. However, this statement has to be tempered from a macroscopic point of view by the observation of strong national disparities, especially in terms of educational requirements and career opportunities.

But even if stereotypes die hard, identity is not set in stone and changes can occur, under the impetus of great leaders such as Florence Nightingale (van der Cingel and Brouwer, 2021) or in the wake of a global event such as the Covid-19 pandemic (Hoseini Azizi et al., 2024).

In the context of a profession that is still in a state of evolution and development, as is the case with advanced practice in France, it is imperative to provide support for the formation of its professional identity. The construction of professional identity is influenced by a focus on socio-cultural contexts; in the case of advanced practice in France, this is further complicated by the nation's unique historical and cultural heritage, which has deeply influenced its nursing history. Initially, advanced practice was rooted in the Christian religion and was subject to the authority of physicians, which has had a significant impact on its development. It is important to acknowledge the historical development of the nursing profession in France, where proper nurse role was formally acknowledged for the first time in 1978.

In addition, nurses seem to have a strong expectation for autonomy, especially in making clinical decisions (Pursio et al., 2021). This need is particularly met by advanced practice nurses, possibly even more so in the ER.

## 3 The study

### 3.1 Aims

The primary aim of this study was to examine the perceived characteristics of the professional identity of emergency nurse practitioners. The secondary objectives were to highlight whether a longer period of collaboration would facilitate a convergence of vision between emergency nurse practitioners and the teams in which they work regarding the latter’s roles and missions. The study also aimed to identify barriers and levers specific to ER, as identified by professionals.

## 4 Methods

### 4.1 Study protocol

A multicenter qualitative study was conducted to investigate the professional characteristics perceived by ER staff (nurses, healthcare managers, physicians, interns) and emergency nurse practitioners themselves regarding the professional characteristics of the emergency nurse practitioner.

### 4.2 Sampling

The snowball sampling technique was employed, starting with the emergency nurse practitioners. Six emergency nurse practitioners were interviewed in five different hospitals. Each emergency nurse practitioner nominated the names of nurses, physicians, and health managers in their ER to participate in the study.

Inclusion criteria were as follows: participants were required to have worked in an emergency department with an emergency nurse practitioner.

### 4.3 Data collection

To construct the semi-directive interview guide for the study, exploratory interviews were conducted based on the existing literature. AC collected the data from April to June 2024. A sociologist (PhD), SG, was implied in the development of the interview guide to benefit as an expert on professional identity. The final interview guide is presented in the table below (Table 1).

**Table 1:** Interview guide.

1	Could you please describe the nature of work performed by ENP?
2	Describe the typical workday of an emergency nurse practitioner.
3	Do you believe that these tasks are shared with other trades?
4	How does the emergency nurse practitioner profession differ from other emergency professions?
5	What are the professional qualities required of an emergency nurse practitioner?
6	Do you perceive emergency nurse practitioner as a threat or an opportunity for your profession?
7	What are your expectations when working with an emergency nurse practitioner?
8	What should an emergency nurse practitioner do that s/he doesn't currently do?
9	What should an emergency nurse practitioner avoid doing?
10	Do you believe that the emergency nurse practitioner profession is uniquely clinical?

AC between February and April 2024 conducted in person or by telephone the interviews. A voice recording was made with the consent of the participants for purpose of transcription. Each interview lasted between 22 and 49 min. The participants were not told about the exact questions they would be asked.The participants were informed of the general topic under discussion, namely the professional identity of the emergency nurse practitioner.

### 4.4 Ethical considerations

This study received approval from the Ethics Committee (CE-2024–28, University of Strasbourg, France, February 28, 2024). All participants gave informed consent, and their data were anonymized. They were informed that their comments would be transcribed and might be cited in the study.

The study complies with current European regulations, and the data were handled according to the General Data Protection Regulation (GDPR). It is also registered with the French data protection authority.

### 4.5 Data analysis

AC analyzed the data under the supervision of TP, emergency physician and holder of a Ph.D. in educational sciences. Thematic analysis was employed (Kiger and Varpio, 2020) with a continuous thematic approach leading to the construction of a thematic tree. Subsequently, selective coding was conducted by LB who does not practice in the emergency field, in order to facilitate distance and ensure the reliability of the coding process. An initial coding was conducted by AC. A representative selection of the 30 % sample was then given to LB for re-coding to ensure that the codes matched. The interviews selected for coding were randomly selected from each of the respondents' professional categories.

The thematic tree was built using a logbook. This process involved noting the interview conditions, identifying themes during analysis, and merging or splitting themes as needed. The researchers also explained their reasoning for these groupings and provided detailed descriptions of each theme's characteristics. Throughout data collection, they refined the interview guide based on ongoing analysis.

Additionally, the interviews were analyzed in terms of both the professions and the hospitals from which the participants originated.

The operational method (Forero et al., 2018) was employed to determine that data saturation had been reached. We concluded that data saturation had been achieved when no new codes were identified during several consecutive interviews. Once it had been determined that data saturation had been reached, the recruitment process was terminated.

### 4.6 Methodological rigor

To ensure the rigor of this study, three main criteria were selected (Forero et al., 2018): credibility, transferability, and reliability.

#### 4.6.1 Credibility and transferability

Researcher triangulation was used to ensure credibility, through co-coding.

For transferability, the data sources were triangulated through interviews with health professionals from five distinct hospitals. The hospitals in question exhibited considerable heterogeneity in terms of size and location. The hospitals in question were of two distinct types: some were large, regional hospitals, while others were peripheral hospitals. Furthermore, the professionals interviewed represented four distinct professions.

A diversity of perspectives is facilitated by the presence of RNs, healthcare managers, physicians and emergency nurse practitioners. Within each profession, further subdivisions were made according to the profile of the respondents. In the case of health managers, some were senior health managers, while others were local health managers. In the case of physicians, the study also included physician trainees. In the case of RNs, those with varying degrees of professional experience were sought. The objective was to create this variety of profile in order to obtain the most extensive range of samples and to guarantee the diversity of profiles.

Additionally, as previously indicated, the point of data saturation was reached.

It can thus be concluded that both credibility and transferability were respected.

#### 4.6.2 Reliability

Given the recent establishment of this profession in France, AC had previously known four of the six emergency nurse practitioners interviewed, as well as one emergency physician, which may have influenced the responses given or their interpretation (prior knowledge bias).

Finally, this study was conducted in accordance with the COREQ criteria for quality assurance (Tong et al., 2007).

## 5 Findings

### 5.1 Population

A total of 21 professionals were interviewed, including seven RNs, three physicians, six emergency nurse practitioners, and five healthcare managers (Table 2) (Table 3). The survey included twice as many women (*n*= 14) as men (*n*= 7) participated in the survey.

**Table 2:** Socio-demographic characteristics of participants.

Participant no.	Hospital	Type	Age range	ENP time in service (+ or - 1 year)	Notes
MAN_1	CH_2	Men	45–55	–	Not the ENP direct supervisor (department manager for over 20 years)
MAN_2	CH_2	Woman	35–45	–	Not the line manager (present in the department for <6 months)
MAN_3	CH_5	Woman	45–55	+	Line manager
MAN_4	CH_1	Men	35–45	+	
MAN_5	CH_3	Woman	45–55	+	Senior health executive
RN_1	CH_2	Woman	18–25	–	6 months emergency room experience
RN_2	CH_2	Woman	45–55	–	20 years' experience in the emergency department
RN_3	CH_2	Men	35–45	–	15 years' experience in the emergency department
RN_4	CH_1	Woman	35–45	+	7 years' experience in the emergency department + plans to become an ENP
RN_5	CH_1	Woman	35–45	+	7 years' experience in the emergency department
RN_6	CH_3	Woman	25–35	+	3 years emergency room experience
RN_7	CH_5	Woman	/	+	/
ENP_1	CH_2	Woman	35–45	–	
ENP_2	CH_5	Woman	45–55	+	
ENP_3	CH_3	Woman	35–45	+	
ENP_4	CH_4	Men	25–35	–	
ENP_5	CH_3	Woman	45–55	–	
ENP_6	CH_1	Men	25–35	+	
MED_1	CH_2	Men	35–45	–	
MED_2	CH_5	Men	45–55	–	
MED_3	CH_1	Men	25–35	+	Resident

(The following abbreviations correspond to the following professions MAN: Manager, RN: Register Nurse, ENP: Emergency Nurse Practitioner, MED: Emergency Physician, CH: Hospital Center).

**Table 3:** Distribution of participants by hospital center.

CH_1						
RN_4	RN_5	MED_3	MAN_4	ENP_6		
CH_2						
RN_2	RN_3	ENP_1	MAN_1	MAN_2	MED_1	
CH_3						
RN_6	ENP_3	ENP_5	MAN_5	RN_6		
CH_4						
ENP_4						
CH_5						
RN_7	ENP_2	MAN_3	MED_2			

Half of the participants had worked with an emergency nurse practitioner for less than a year (*n* = 10), while the other half had worked with at least one emergency nurse practitioner for more than a year (*n* = 11).

The RNs were divided into three experience groups: those with less than three years of experience (*n* = 2), those with between three and ten years of experience (*n* = 2), and those with >10 years of experience (*n* = 2). Length of experience and age range were not collected for one nurse.

ENP_4 encountered challenges in establishing contact with professionals from its team, which prevented researchers from being put in touch with them. In all the other hospitals, all professional groups are represented, with the exception of CH_3, where it was not possible to establish contact with an emergency physician.

### 5.2 Themes

The coding process yielded four primary themes. These themes and their corresponding categories and subcategories are presented in Table 4.

**Table 4:** Summary of themes and their categories.

THEME 1: A combination of skills in a variety of fields	
Categories	Subcategories
POLYVALENCE	Trivalence: intra-hospital, pre-hospital, regulation
	Cross-disciplinary skills
	Advanced clinical expertise
COMBINED SKILLS	Integrates skills shared with other professions (e.g. management skills with executives)
	Prescription
	Diagnosis
**THEME 2: A pivotal professional, expert in the care pathway, at the interface with other emergency professions**	
**Categories**	**Subcategories**
TRAIL	Channel
	Coordination
	Patient discharge
	Orientation
	Flow management
	Knowledge of the working environment and healthcare system: Networks
	Integrates the notion of temporality / speed
GRADATION OF RESPONSE TO DEMAND FOR CARE	Intermediate skills
	Graduated response to demand for care
INTERPROFESSIONAL COLLABORATION	Interprofessional collaboration
	Communication
**THEME 3: A dual identity for nurses: nurses and emergency professionals**	
**Categories**	**Subcategories**
NURSING KNOWLEDGE	Basic nursing knowledge
	Holistic dimension
	Technical skills
	Interpersonal skills
SPECIFIC FEATURES OF EMERGENCY SERVICES	Difference with other mentions
	Issues specific to emergency services
	Necessary qualities for emergency professionals
LEADERSHIP	Contact
	Clinical leadership
	Knowledge & skills transfer
**THEME 4: A position that is still in its nascent stages, beset with uncertainty and obstacles.**	
**Categories**	**Subcategories**
RELATIVE AUTONOMY	Under medical cover
	Incorporates the notion of a desire for greater autonomy
	Notion of responsibility
UNCERTAINTY GENERATED BY NOVELTY	New
	Uncertainty

#### 5.2.1 A combination of skills in a variety of fields

The analysis of this theme reveals two main categories: versatility and combined skills. In France, it is possible to work as a nurse or physician in variety of settings, including pre-hospital, in-hospital, and medical dispatch settings. Twelve participants proposed the possibility of achieving this threefold role for the emergency nurse practitioner.

"The emergency nurse practitioner has a role to play in pre-hospital and in emergency medical dispatch." (MAN_4).

As evidenced by MAN_4, the ability to perform the duties of a nurse is also a desirable quality in an emergency nurse practitioner.

The versatility of roles was also highlighted in the potential for different positions within in-hospital emergencies. However, this phenomenon was less pronounced than in the case of the resuscitation room, for instance (*n*= 5).

"I could see her working at the resuscitation room reinforcing the paramedical team on site" (ENP_5)

Versatility was also mentioned as a cross-cutting skill for the emergency nurse practitioner. The participants mentioned team training (*n*= 14) and aspects of nursing research (*n*= 17).

"It is accurate to state that there is often a lack of time to conduct research in the context of care. However, the emergency nurse practitioner has received training in this area throughout her career, and therefore, it is possible to allocate time for her to engage in research that will enhance our practices." (MAN_5).

With regard to the skills that the emergency nurse practitioner shares with other professions, two other professions were mentioned in particular: physicians and healthcare managers. About physicians, the competencies include those related to clinical examination, the right to prescribe (in particular, additional tests), and the formulation of diagnostic hypotheses.

"She would install the patients, perform a clinical examination, and based on the diagnostic hypotheses, prescribe additional tests, interpret them, and then advance the case as far as she could" (MED_1).

Regarding skills shared with managers, both the managers and emergency nurse practitioners interviewed agreed that emergency nurse practitioners do not handle managerial tasks related to human resources, such as scheduling or staff evaluations. However, everyone acknowledged the potential for collaborative work, especially in enhancing team skills. Importantly, no manager expressed feeling threatened by the presence of an emergency nurse practitioners on their team.

"The only area in which we might have some common ground is in terms of the analysis of professional practices" (ENP_5).

#### 5.2.2 A pivotal professional, expert in the care pathway, at the interface with other emergency professions

Participants discussed the role of the emergency nurse practitioner in the patient pathway, as well as the intermediate response in the care offering it represents.

Most participants acknowledged the emergency nurse practitioner's expertise in the patient pathway. The initial aspect to be considered is the coordination of intra-hospital care.

"Care is often segmented, so we are able to link all relevant parties together. Furthermore, the development of the care project sets us apart" (ENP_2).

A second area of expertise is the emergency nurse practitioner's ability to facilitate the patient pathway through the emergency department.

"They are intimately familiar with the patient pathway, having experienced it from the outset of care, from initial reception to discharge. They were first nurses in the emergency department, which allows them with a deep understanding of the patient journey" (RN_4).

A third point involves setting up communication channels and coordinating all healthcare professionals around a patient in the emergency department.

"Our objective is not to supplant them, but rather to elucidate their role, to establish a connection between the patient and the other elements that are lacking" (ENP_1).

A fourth aspect pertains to the establishment of a link between the city hospital and other healthcare facilities.

"I believe that the emergency nurse practitioner could serve as a conduit for discussion and care, a conduit that would facilitate communication between the hospital and external care providers" (RN_2).

Finally, a fifth aspect underscores the necessity for coherence within the care pathway, with the aim of reducing the number of repeat visits to emergency departments.

"It is necessary to propose care pathways that are coherent and to avoid people who are seen in the ER repeatedly because they have not found a solution." (MED_2)

Regarding the intermediary care between physicians and nurses, this was mentioned by the majority of participants (*n*= 15), including physicians (*n*= 2).

"I think she's a bit of a cross between a nurse and a physician" (MAN_2)

A notable trend emerged among participants in their comparison of the role of the emergency nurse practitioner to that of resident

"Even if we know it's not a resident, in the end it's still similar" (MAN_5)

For some, they point to at least comparable (*n*= 13) or even more pronounced expertise on the part of the emergency nurse practitioner than the intern (*n*= 9).

"I do not perceive any distinction between the roles of the resident and the emergency nurse practitioner" (MED_3)

"In practical terms, I am more confident in my emergency nurse practitioner than in the resident" (MED_2)

Some participants (*n* = 8) have noted that the emergency nurse practitioner is a qualified professional, whereas the resident is still a professional in training.

"Residents are physicians in training, while the emergency nurse practitioner is a qualified professional" (MAN_1).

It is also worth noting that some participants (*n* = 4) noted that the presence of emergency nurse practitioners improved the quality of resident training.

#### 5.2.3 A dual identity: nurse and emergency professional

The emergency nurse practitioner is perceived as both a nurse and an emergency expert. There are notable divergences in this regard, particularly about the nurses' strong identity anchoring.

" It's true that it's silly, but nursing is often linked to taking blood tests, and I don't see her taking blood tests now.” (RN_1).

"The emergency nurse practitioner is no longer a nurse in the traditional sense" (MAN_1).

Conversely, for others, the emergency nurse practitioner is perceived as a nurse who has developed more advanced competencies. For the participants, the emergency nurse practitioner is an advanced nurse who is able to prescribe and perform a more comprehensive clinical examination.

The majority of participants identified two primary distinguish characteristics between the emergency nurse practitioner and the physicians: interpersonal skills and holistic patient management.

"There's a difference with physicians in the way they treat patients, let's say in a humane way, because we have the same basic profession, which in my opinion is much more relational than medical training." (RN_4).

"The emergency nurse practitioner will see the whole patient, more than a physician who will only focus on the disease. For example, if the patient comes in with an arm deformity, the physician will only see the arm deformity, whereas the emergency nurse practitioner will see the whole patient.” (RN_5)

The physicians do not discuss these specifics. The physicians only mention the technical aspects of the emergency nurse practitioner and the severity of the medical conditions treated (*n*= 3).

Regarding the specific features of the ER, two elements are highlighted: The management of patients with acute pathologies (*n*= 9) and the management of a wide range of medical conditions (*n*= 6).

"For me it is different in the sense that it can take care of patients who are decompensated, compared to other specializations that only work on stable patients." (ENP_2).

"There is no target pathology, and one must be proficient in all areas" (ENP_1).

The emergency nurse practitioner is perceived as a potential leader within the emergency department (*n*= 11).

"The emergency nurse practitioner has brought a breath of fresh air back into the ER and is lifting it to new heights" (RN_5).

"The manager is the one who coordinates care, the leader is the one who goes a long way to improve care." (MAN_5).

Nevertheless, it is notable that some participants have indicated that leadership is not a characteristic exclusive to the emergency nurse practitioner.

" As far as leadership is concerned, you don't have to be an emergency nurse practitioner to claim it! " (MAN_4).

#### 5.2.4 A position that is still in its nascent stages, beset with uncertainty and obstacles

The relative autonomy of emergency nurse practitioners was noted, as were the novelty aspects and obstacles mentioned by the participants.

With regard to autonomy, the majority of participants indicated that emergency nurse practitioners are unable to function without the presence of a physician.

«Yes, they have superior knowledge to RNs, but they are not physicians, and without a physician they cannot practice." (MAN_3)

Some participants emphasized the lack of autonomy, particularly regarding emergency nurse practitioners’ prescribing authority (*n*= 9).

"The only thing I hope for is for you to have more autonomy. To give value to emergency nurse practitioners, you need to be free to manage your own case. I'm a physician, and I don't think I have a monopoly on treating people." (MED_2).

Nevertheless, the majority of emergency nurse practitioners expressed satisfaction with their autonomy (*n*= 5).

“In terms of autonomy, it's pretty much what I expected.” (ENP_2)

They also mention the newness of the job, which creates uncertainty:

“It's very complicated, I really have a hard time understanding their positioning.” (RN_7)

“It's difficult because for the "emergency" section, everything has yet to be created.” (MAN_5)

The uncertainty appears to dissipate with the passage of time spent working with an emergency nurse practitioner. As one participant noted:

"His role has become much more precise since I started working with him" (MAN_4).

Finally, the participants spontaneously mentioned some of the obstacles they encountered during the implementation process. These included resistance from the nursing team (*n*= 3) and funding and salaries (*n*= 2).

"There is a kind of divide that can exist between nurses" (MAN_3).

«You have a lot more responsibility, but you don't get paid more than an RN for doing the job." (RN_1).

## 6 Discussion

The objective of this study was to ascertain the professional characteristics of the emergency nurse practitioners. It was found that the perceived characteristics of the emergency nurse practitioner are consistent with the competencies described by Hamric (Tracy et al., 2023). These include clinical practice, evidence-based practice through research and analysis of professional practices, leadership, and collaboration, as reflected in the notion of the pivotal professional coordinating care pathways. The issue of ethics was not addressed by the participants. A closer look at clinical practice shows that among the six traits Hamric describes are a holistic approach, training, clinical expertise, and evidence-based nursing (Tracy et al., 2023). We can assume that the training of emergency nurse practitioners reflects their foundational thinking model, as explained by the Hamric model.

The mixed skills of the emergency nurse practitioner have been a significant focus of the study, whether we are discussing a combination of skills or a combination of practice locations. This appears to be a distinctive feature of emergency medicine.

Moreover, the participants rarely mentioned the physician’s shortage, as the emergency nurse practitioner's contribution is more focused on the notion of a care pathway and holistic patient care. It is crucial to acknowledge that the potential for conflict with the care team was already apparent, and it appears that the emergency nurse practitioner is not exempt from this.

In Schwingrouber's study, one of the risks identified was the perception of advanced practice nurses as "*sub-physicians, sub-resident, a kind of resident*" (Schwingrouber et al., 2021). It was observed that, in terms of clinical skills, emergency nurse practitioners in ER were considered to be equivalent to medical professionals still in training, with a view that was sometimes considered to be more acute with regard to management. Furthermore, it has been observed that economic constraints continue to affect emergency nurse practitioners. In France, advanced practice nurses continue to face significant economic challenges, including limited financial recognition and barriers to sustainable remuneration. These issues hinder the full implementation of their roles within the healthcare system.

Regarding nursing identity, while interviewed emergency nurse practitioners affirm their status as full nurses, RNs often see them as no longer true nurses. Those holding this view cite the decline in procedural care as the reason emergency nurse practitioners are not seen as fully nurses. However, emergency nurse practitioners strongly disagree, highlighting that their unique nursing perspective enhances medical care quality. Indeed, environmental awareness is central to emergency nurse practitioner discourse. The emergency nurse practitioner profession is based on the nursing meta-paradigm, which outlines the overall perspective and scope of the discipline. This meta-paradigm includes four key concepts: care, the person, health, and the environment (Fawcett, 1984). The surveyed emergency nurse practitioners particularly emphasize care and environment and align with Girard and Cara's humanist nursing model, characterized by eight competencies: 1) Practicing humanism from a disciplinary perspective, 2) Applying nursing clinical reasoning, 3) Ensuring care continuity, 4) Promoting population health, 5) Approaching all activities with scientific rigor, 6) Acting professionally, 7) Collaborating in teams, 8) Demonstrating clinical leadership in nursing practice (Cara et al., 2016).

This perspective on nursing clinical reasoning has some limitations. Nurses in the emergency nurse practitioner program are seen as having an intermediate role in the French healthcare system. They can manage patients independently but still under physician supervision. This partial autonomy is somewhat paradoxical, as emergency nurse practitioners often assume they need medical oversight. Meanwhile, physicians in our study question the need for this supervision, suggesting it may hinder care.

This partial autonomy creates ambiguity about the emergency nurse practitioners' role. The paradox may stem from French nurses' recent move towards independence from medical supervision. The French healthcare model is primarily structured around medical practice, with paramedical roles often requiring medical approval (Benoit, 2019). Moreover, some of the RNs in our study observed that emergency nurse practitioners no longer have a prescribed role, in contrast to RNs, because they are themselves prescribers.

Further investigation is warranted into the concept of intermediary and interface, both in the gradation of pathologies managed and in the echelon represented by the emergency nurse practitioner profession. We describe this skill as "*mesopraxia*," a neologism derived from the Greek prefix "*meso*" (situated in the middle) and the Latin noun "praxis" (method, technique, practice). The term aims to emphasize the intermediate role of the emergency nurse practitioner. However, the concept of an intermediary doesn't fully capture the range of skills in the emergency nurse practitioner profession. Emergency nurse practitioners possess a broad set of nursing skills, enhanced by specific medical abilities, raising the question of whether the profession is better described as hybrid.

In her 2020 work, Gabrielle Halpern, a French philosopher, develops the notion of hybridization in an appropriate manner, employing the mythological image of the centaur, half-man, half-horse, which does not fit into either the conceptual boxes of man or horse (Halpern, 2020). Rather, it occupies a third category. As Halpern (2020) notes, we are witnessing a veritable "hybridization of professions." Hybridization is defined ‘*as neither fusion nor juxtaposition, neither assimilation nor annihilation of the other, but rather as the metamorphosis of each. This is only possible if each accepts to step out of their own identity in order to take a step towards the other.’* (Halpern, 2020)(translation by the authors).

### 6.1 Strengths and limitations

The research team achieved data saturation and achieved satisfactory triangulation. However, the fact that the majority of emergency nurse practitioners were known by AC represents a potential limitation. In addition, participants did not have access to the final transcripts. Finally, we can emphasize that there were no divergent cases highlighted by the interviews, probably due to the size of the sample or the fact that the participants were particularly interested or concerned about the issue.

Nevertheless, one of the major strengths of this study is that it focuses on emergency teams, which to our knowledge has not been done before, despite the working environment of emergency departments.

Finally, given the relative youth of the profession in France and the high expectations that surround it, there is a possibility that a social desirability bias may have influenced the responses.

Moreover, the distinctive characteristics of the French system necessitate the implementation of a more extensive study in other regions. A French study published in 2021 found that the characteristics of French advanced practice nurses were strongly similar to the professional characteristics described by the International Council of Nursing for the advanced practice nurse profession, with the exception of diagnosis and primary access (Colson et al., 2021) what is currently being resolved at regulatory level.

### 6.2 Implications for practice and future research

Future research is needed to explore the concept of hybridization to confirm its relevance in conceptualizing advanced nursing practice in emergency departments. Additionally, the ideas of intermediary roles and gradation in emergency settings require further study.

This study helps students and managers understand the expected traits and potential challenges of implementing emergency nurse practitioners in emergency services. Further research is necessary to determine if the unique aspects of the French context affect these challenges and the implementation model.

Finally, this study provides valuable insights for students and educators advanced practice nurses' training, highlighting the expected characteristics of future professionals. These insights can guide the development of training and working methods that align with these traits.

## 7 Conclusion

This study reveals that the emergency nurse practitioners' professional characteristics align with Hamric's model, which serves as the foundation for advanced practice in many countries worldwide. Nevertheless, some specificities emerged in relation to previous research on advanced practice nurses' perceptions.

Furthermore, the emergency nurse practitioner represents a novel presence within the healthcare system and is regarded by most as a pivotal figure in enhancing patient care in emergency departments.

Finally, new concepts are emerging that warrant further investigation, including hybridization and intermediary, as well as the concept of "*mesopraxia*."

## Declaration of generative AI and AI-assisted technologies in the writing process

During the preparation of this work the author(s) used OpenAI tool in order to improve the language. After using this tool, the authors reviewed and edited the content as needed and takes full responsibility for the content of the publication.

## Ethics approval statement

Approval was obtained from the local ethics committee (University of Strasbourg, CE-2024–28, February 28, 2024).

## Patient consent statement

There were no patients included in this study. Oral consent to record and transcribe the interviews was obtained for each participant. The study was registered with the French National Agency regulating Data Protection.

## Permission to reproduce material from other sources

Not applicable, the manuscript is not published elsewhere.

## Clinical trial registration

Not applicable.

## Funding

This research did not receive any specific grant from funding agencies in the public, commercial, or not-for-profit sectors.

## CRediT authorship contribution statement

**Aline Chenou:** Writing – review & editing, Writing – original draft, Methodology, Investigation, Formal analysis, Conceptualization. **Loïc Brillouet:** Investigation, Formal analysis. **Stéphane Guillon:** Resources. **Pascal Bilbault:** Writing – review & editing, Supervision. **Thierry Pelaccia:** Writing – review & editing, Supervision, Resources, Methodology, Conceptualization.

## Declaration of competing interest

The authors have no conflict of interests related to this publication.
